# Expression Profiles of Fsh-Regulated Ovarian Genes during Oogenesis in Coho Salmon

**DOI:** 10.1371/journal.pone.0114176

**Published:** 2014-12-08

**Authors:** José M. Guzmán, J. Adam Luckenbach, Yoji Yamamoto, Penny Swanson

**Affiliations:** 1 Environmental and Fisheries Sciences Division, Northwest Fisheries Science Center, National Marine Fisheries Service, National Oceanic and Atmospheric Administration, Seattle, Washington, 98112, United States of America; 2 Center for Reproductive Biology, Washington State University, Pullman, Washington, 99164, United States of America; 3 School of Aquatic and Fishery Sciences, University of Washington, Seattle, Washington, 98195, United States of America; 4 Department of Marine Biosciences, Tokyo University of Marine Science and Technology, 4-5-7 Konan, Minato-ku, Tokyo, 108-8477, Japan; Inner Mongolia University, China

## Abstract

The function of follicle-stimulating hormone (Fsh) during oogenesis in fishes is poorly understood. Using coho salmon as a fish model, we recently identified a suite of genes regulated by Fsh in vitro and involved in ovarian processes mostly unexplored in fishes, like cell proliferation, differentiation, survival or extracellular matrix (ECM) remodeling. To better understand the role of these Fsh-regulated genes during oocyte growth in fishes, we characterized their mRNA levels at discrete stages of the ovarian development in coho salmon. While most of the transcripts were expressed at low levels during primary growth (perinucleolus stage), high expression of genes associated with cell proliferation (*pim1*, *pcna*, and *mcm4*) and survival (*ddit4l*) was found in follicles at this stage. The transition to secondary oocyte growth (cortical alveolus and lipid droplet stage ovarian follicles) was characterized by a marked increase in the expression of genes related to cell survival (*clu1*, *clu2* and *ivns1abpa*). Expression of genes associated with cell differentiation and growth (*wt2l* and *adh8l*), growth factor signaling (*inha*), steroidogenesis (*cyp19a1a*) and the ECM (*col1a1*, *col1a2* and *dcn*) peaked in vitellogenic follicles, showing a strong and positive correlation with transcripts for *fshr*. Other genes regulated by Fsh and associated with ECM function (*ctgf*, *wapl* and *fn1*) and growth factor signaling (*bmp16* and *smad5l*) peaked in maturing follicles, along with increases in steroidogenesis-related gene transcripts. In conclusion, ovarian genes regulated by Fsh showed marked differences in their expression patterns during oogenesis in coho salmon. Our results suggest that Fsh regulates different ovarian processes at specific stages of development, likely through interaction with other intra- or extra-ovarian factors.

## Introduction

Oogenesis is the developmental process by which an oogonium becomes a fully-grown, mature, and fertilizable egg. As in other vertebrates, oogenesis in fishes is regulated primarily by pituitary gonadotropins, follicle-stimulating hormone (Fsh) and luteinizing hormone (Lh), through interaction with their respective receptors, Fsh receptor (Fshr) and luteinizing/choriogonadotropin receptor (Lhcgr), in the ovary [1], [2]. Most studies of the biological activities of fish gonadotropins have focused on Lh regulation of sex steroid biosynthesis and final oocyte maturation [3], [4], whereas relatively few studies have investigated how Fsh may regulate oocyte growth and development during earlier stages of oogenesis [2], [5].

Coho salmon (*Oncorhynchus kisutch*) is a semelparous species (i.e., spawns only once in its life and then dies) that exhibits synchronous follicle development. This unique reproductive life history makes coho salmon an excellent model for the study of specific stages of oogenesis, because within the ovary, at any given time, the follicles are typically homogeneous and gene expression results are not confounded by the presence of other stage follicles. The developmental profile of Fsh is also well characterized in coho salmon. Plasma levels of Fsh increase during the transition from primary to early secondary oocyte growth along with an increase in ovarian *fshr* mRNA and plasma sex steroid levels [6], [7]. Subsequently, during vitellogenesis, plasma Fsh continues to rise until just prior to final maturation, at which point Fsh levels decline and Lh levels surge leading up to ovulation [8], [9]. These findings suggest that Fsh plays an important function from at least the onset of the early secondary oocyte growth until the completion of vitellogenesis, and this supposition is supported by data in other species [10], [11], [12], [13].

It is well documented that during secondary oocyte growth, the developing ovarian follicles undergo massive structural and functional changes. These include synthesis of cortical alveoli (formerly yolk vesicles), increased potential for steroid production and accumulation of lipids and yolk proteins from the blood, followed by massive growth of the oocyte [14], [15]. At that time, numerous intrafollicular autocrine and paracrine mechanisms are also established, and the oocyte completes the formation of the egg envelope [2], [16]. Although the transition through these stages is critical for puberty onset, egg quality, and further embryo development, the role of Fsh during this period is only starting to be revealed.

Two recent studies have identified ovarian genes regulated by Fsh in vitro during early secondary oocyte growth in coho salmon. In the first study, Fsh-regulated ovarian genes were identified through a candidate gene approach [17]. We found that Fsh regulated specific steroidogenesis-related genes (e.g., *star* and *hsd3b*) and had a strong stimulatory effect on estradiol-17β (E2) production. Furthermore, Fsh also altered mRNA levels of gonadotropin receptors (*fshr* and *lhcgr*), transforming growth factors (e.g., *bmp16*) and an anti-apoptotic factor (*clu1*), suggesting that besides the established role on ovarian steroidogenesis, Fsh also regulates genes associated with ovarian cell growth, differentiation and survival. In the second study we used a more global transcriptome analysis technique known as suppression subtractive hybridization to identify a broader range of ovarian Fsh-regulated genes [18]. In this study, we demonstrated that Fsh regulates the in vitro expression of a unique suite of genes involved in a number of ovarian processes mostly unexplored in fishes, like cell survival (e.g., *clu2*, *ivns1abpa*), proliferation (e.g., *pim1*, *pcna*), differentiation and growth (*wt2l*, *adh8l*), tissue and extracellular matrix (ECM) remodeling (e.g., *ctgf*, *wapl*).

In mammals, a large body of evidence indicates that FSH is essential for normal acquisition of puberty and fertility [19], and FSHβ knockout female mice show inhibition of follicular growth, a small uterus and are sterile [20]. In mammals it is also well established that FSH drives oocyte growth via modulation of the expression of a variety follicle cell and oocyte factors throughout folliculogenesis [21], [22].

We now have a better understanding of major Fsh-regulated ovarian genes in salmonids; however, gene expression responses to a single hormone in vitro can differ greatly from an in vivo gene expression profile. Aside from gonadotropin receptors and genes encoding steroidogenic enzymes [23], [24], [25], data on the temporal profiles of mRNAs for genes regulated by Fsh throughout fish oogenesis are lacking. This study characterizes temporal changes in mRNA levels of the recently identified suite of Fsh-regulated genes in coho salmon, focusing on discrete stages of oogenesis including primary growth (perinucleolus stage), secondary growth (cortical alveolus, lipid droplet and vitellogenic stages) and final maturation.

## Materials and Methods

### Samples, RNA isolation and cDNA synthesis

To characterize the expression profiles of Fsh-regulated genes in coho salmon during oogenesis, we used ovarian follicle cDNAs previously employed to determine the expression patterns of connexin genes [26]. In brief, hatchery-produced age-1+ and -2+ coho salmon were reared at the Northwest Fisheries Science Center (Seattle, WA, USA) and sampled to obtain follicles at different stages of ovarian development: perinucleolus (PN)-stage, cortical alveolus (CA)-stage, lipid droplet (LD)-stage, mid-vitellogenic (VIT)-stage and postvitellogenic/preovulatory (MAT)-stage. Prior to tissue sampling, fish were euthanized in buffered tricaine methanesulfonate (0.05% MS-222, Argent Chemical, Redmond, WA).

Total RNA was isolated from pieces of ovarian tissue (PN, CA, LD and VIT stages, n = 4 fish/stage) or 5 follicles/fish (MAT-stage, n = 3 fish) using Tri-reagent (Molecular Research Center, Cincinnati, OH) and DNase treated using the Turbo DNA-Free kit's rigorous protocol (Life Technologies, Austin, TX). Messenger RNA was further isolated from total RNA samples using the MicroPoly(A)Purist kit (Ambion, Austin, TX). It was not possible to count ovarian follicles at all stages of oogenesis, therefore transcript levels could not be expressed on a per follicle basis. However, previous work in coho salmon demonstrated that using mRNA as template and normalizing to reference genes for quantitative PCR generates results that best reflect transcript abundance on a per follicle basis [7]. For cDNA synthesis, 50 ng of mRNA was reversed transcribed with the Superscript II kit (Invitrogen, Carlsbad, CA). Other necessary components for the reverse transcription (RT), such as random primers and RNase inhibitor, were purchased from Promega (Madison, WI). Negative control reactions were performed without the addition of the RT enzyme for a subset of the RNA samples. Complementary DNA samples were stored at −30°C until use.

### Ethics statement

Fish used in the experiment were reared and handled in strict accordance with the policies and guidelines of the University of Washington Institutional Animal Care and Use Committee (IACUC Protocol #2313-09) which specifically approved this study.

### Quantitative real time RT-PCR (qPCR)

The qPCR methods were previously described [17]. Briefly, qPCR assays were run on an ABI 7900HT Fast Real-Time PCR System (Life Technologies) in 384-well plates using standard cycling conditions: 50°C for 2 min, 95°C for 10 min, followed by 40 cycles of 95°C for 15 s and 60°C for 1 min. Reactions were 12.5 µl each and consisted of 1X Power SYBR Green master mix (Applied Biosystems), 150 nM of the forward and reverse primer (Table 1), and 0.5 ng of cDNA template. For each gene, all samples were assayed in the same plate to avoid across plate variation. Data were normalized using the geometric mean of four reference genes (*eef1a*, *ctsd*, *ctsz* and *actb*) which was stable among major stages of oogenesis in coho salmon (S1 Figure), as previously reported in tilapia [27]. To improve presentation of results, the mean value of the PN-stage was set to 1, so all normalized data are divided by the mean of the PN-stage.

**Table 1:** Targeted Fsh-regulated ovarian genes analyzed by qPCR during oogenesis in coho salmon, type of regulation by Fsh, qPCR primers and PCR product size.

	Symbol	Regulation by Fsh in vitro a	Forward Primer	Reverse Primer	Product size (bp)
**Gonadotropin receptors**					
*follicle-stimulating hormone receptor*	*fshr*	Down-regulated	gacgcacatcagagtgtttccc	gtagaaccctcagtccagtgttgc	242
*luteinizing/choriogonadotropin receptor*	*lhcgr*	Up-regulated	tatccattctctggaaccttgg	cttggtcccattaaaggcatag	175
**Steroidogenesis-related genes**					
*cytochrome P450, subfamily XIA, polypeptide 1*	*cyp11a1*	Up-regulated	tcatggtgcacaacttcaacac	gttcctgtagtctctgtatga	173
*cytochrome P450, family 17, subfamily A, polypeptide 1*	*cyp17a1*	Up-regulated	agagacaagctgcttcagaa	gcccattttaggactgttgacg	230
*cytochrome P450, family 19, subfamily A, polypeptide 1a*	*cyp19a1a*	No effect	acccgcacctacttcgctaaag	tgctctcctgtgtttctgctgg	315
*hydroxy-5-steroid dehydrogenase, 3 beta*	*hsd3b*	Up-regulated	ccttcatctacaccagcagcatc	tacaacacatccccgttccg	283
*steroidogenic acute regulatory protein*	*star*	Up-regulated	gggacttcgttagtgttcgctg	tggtcttgttggggtcatcg	168
**Cell survival-related genes**					
*clusterin 1*	*clu1*	Up-regulated	aggacctctccattctccatctg	gccatctctgctctctcattgg	260
*clusterin 2*	*clu2*	Up-regulated	caggccctagacctctacaaac	gaagtcatcctgaacattctgc	104
*influenza virus NS1A-binding protein a*	*ivns1abpa*	Up-regulated	ctgagctgtggggagactta	gtcaaaagcgtcacagttcttc	176
**Extracellular matrix components**					
*collagen alpha 1(I) chain*	*col1a1*	Down-regulated^b^	cataccactgcaagaacagcat	caataataggcagacgggatgt	222
*collagen alpha 2(I) chain*	*col1a2*	Down-regulated^b^	caaccaggctacccagaacatc	tgactgtcttgctccattggc	203
*decorin*	*dcn*	Up-regulated	gaccacaagtacatccaggtga	aacacacagcggaaggtgat	176
*fibronectin*	*fn1*	Up-regulated	gctcttcagaatgtccagagaa	aggccgttgttacctactactg	167
**Tissue or extracellular matrix remodeling-related genes**					
*connective tissue growth factor*	*ctgf*	Down-regulated	ccctaactactcgcaagagact	gtaggagagagtgaggcagaag	151
*whey acidic protein domain containing–like*	*wapl*	Up-regulated	gtggggatgtgtgtggagtt	cacacatgtaggtcccgttg	147
**Cell proliferation-related genes**					
*serine/threonine-protein kinase pim-1*	*pim1*	Down-regulated	tcagacgaagaatctccacaga	tagtctctgtcagtgcagccat	229
*proliferating cell nuclear antigen*	*pcna*	Down-regulated^b^	gaaagttttggaggctctgaagg	gttctgctcacctgctaagattgac	194
*DNA replication licensing factor MCM4*	*mcm4*	Up-regulated^b^	ctgtgctgaaggactacattgc	caatcttcctcatgtccacgta	108
*protein tob1*	*tob1*	Up-regulated^b^	ttagagcagttccacaaagctc	gaagttgagggctacttggatt	206
**Growth factor signaling-related genes**					
*bone morphogenetic protein 16*	*bmp16*	Down-regulated	gaaggagagattgatggcgtaaag	tgctggattgggttctacattcc	181
*insulin-like growth factor 2*	*igf2*	Up-regulated	gacacgaacaccactcagtttgc	cttggcaggtttggcacaatac	329
*inhibin-specific alpha subunit*	*inha*	Up-regulated^b^	tttggaccgcaccactgctatg	actttgaaacactgagcccatctc	197
*mothers against decapentaplegic homolog 5-like*	*smad5l*	Down-regulated^b^	gcacacatcttggtgagttcgtag	tgacagcagtatcttcgtccagag	186
**Cell differentiation and growth-related genes**					
*wilms tumor protein 2-like*	*wt2l*	Up-regulated	aagcgtccgtttgtgtgtgc	ctgtggtggcgaactaactcatc	350
*alcohol dehydrogenase 8-like*	*adh8l*	Down-regulated	cacttgtaaggggaagaaggtg	aggagacagaccttgtcgagag	126
**Reference genes**					
*elongation factor 1 alpha*	*eef1a*	No effect	cccctggacacagagatttcatc	agagtcacaccgttggcgttac	409
*cathepsin D*	*ctsd*	No effect	cgtcatctttgactcccgatcc	gcacaagtttccatttgcttttctg	175
*cathepsin Z*	*ctsz*	Unknown	tcccatcgttcccaaaacctac	tgttcccaaggcaaagcacg	200
*actin*, *beta* c	*actb*	Unknown	ggawgatgaaatygccgcac	trcccatctcctgctcraagtc	683

a Based on studies in cortical alveolus stage ovarian follicles [17], [18].

b Effect of Fsh was only observed in follicle cell enriched preparations.

c Assay validated for coho and Chinook salmon.

### Gene nomenclature

Gene and protein nomenclature of the ZFIN database were used whenever possible. In this database, genes are named using mammalian orthologs but italicized and lower case (e.g., *fshr*) and the protein symbol is the same as the gene symbol, but not italicized, and the first letter is uppercase (e.g., Fshr). When discussing mammalian or mammalian and fish genes and proteins generally, mammalian nomenclature was used (e.g., FSHR). Complete names and symbols of genes analyzed in the present study are shown in Table 1.

### Statistics

Statistical analyses were performed using Prism 5 software for Mac OSX (GraphPad Software, La Jolla, CA) with the minimum level of significance set to P<0.05. Differences were examined using one-way ANOVA and where significant differences were observed, Tukey's Multiple Comparisons Tests were performed. When necessary, data were log or square-root transformed in order to comply with normality and homogeneity of variance. Data are expressed as mean ± standard error of the mean (SEM). Correlation coefficients between parameters were calculated using Pearson's *r*.

## Results and Discussion

In the present study we report expression profiles of Fsh-regulated genes during oogenesis in coho salmon. This suite of genes was recently identified after exposing salmon ovarian follicles at the early cortical alveolus stage to purified native Fsh in vitro [17], [18]. Since most of these genes likely participate in ovarian processes mostly unexplored in fishes, the functional categories in which they are classified in the present study should be considered as only a guide.

### Glycoprotein hormone receptors

**Figure 1:**
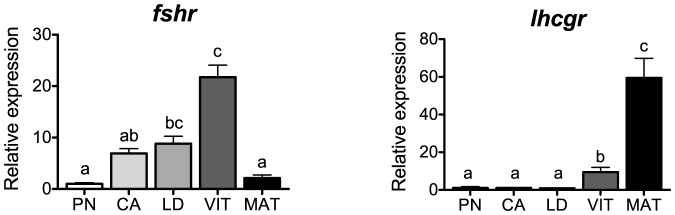
Expression profiles of gonadotropin hormone receptor genes during major stages of oogenesis in coho salmon. Messenger RNA levels were analyzed by qPCR and data were normalized to the geometric mean of four reference genes (*eef1a*, *ctsd*, *ctsz* and *actb*). PN, perinucleolus stage follicles; CA, cortical alveolus stage follicles; LD, lipid droplet stage follicles; VIT, mid-vitellogenic stage follicles; MAT, postvitellogenic/preovulatory stage follicles. Bars not sharing the same letter are significantly different (P<0.05; n = 4 fish/stage for PN-, CA-, LD-, and VIT-stages, n = 3 fish for MAT-stage, mean ± SEM).

Transcript levels of the gonadotropin receptors, *fshr* and *lhcgr*, are shown in Fig. 1. The level of *fshr* transcripts increased during the transition into secondary oocyte growth, peaked in VIT-stage follicles, and declined thereafter. In contrast, levels of *lhcgr* transcripts remained low during previtellogenic stages, increased during vitellogenesis and peaked at the MAT-stage. These stage-specific profiles correlate well with the temporal patterns described by their cognate ligands, Fsh and Lh, at both the pituitary gene expression and pituitary and plasma protein level in coho salmon [8], [9] as well as in other fish species [11], [28], [29], [30]. Our results in coho salmon agree with the proposed role of Fsh during early secondary growth and vitellogenesis, and Lh at final oocyte maturation in fishes [1], [2].

### Steroidogenesis

**Figure 2:**
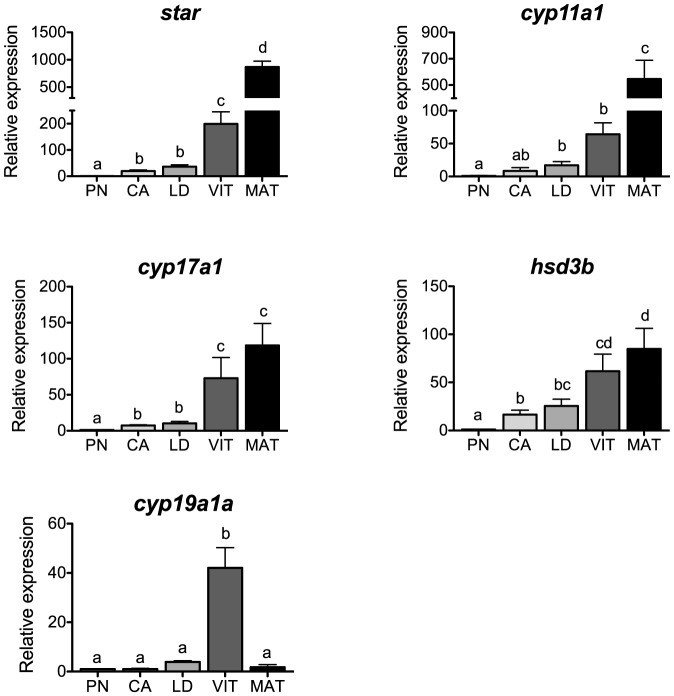
Expression profiles of steroidogenesis-related genes during major stages of oogenesis in coho salmon. Messenger RNA levels were analyzed by qPCR and data were normalized to the geometric mean of four reference genes (*eef1a*, *ctsd*, *ctsz* and *actb*). PN, perinucleolus stage follicles; CA, cortical alveolus stage follicles; LD, lipid droplet stage follicles; VIT, mid-vitellogenic stage follicles; MAT, postvitellogenic/preovulatory stage follicles. Bars not sharing the same letter are significantly different (P<0.05; n = 4 fish/stage for PN-, CA-, LD-, and VIT-stages, n = 3 fish for MAT-stage, mean ± SEM).

Levels of transcripts for the steroidogenesis-related genes *star*, *cyp11a1*, *cyp17a1*, *hsd3b* and *cyp19a1a* are shown in Fig. 2. Levels of *star* and *cyp11a1* were lowest at the PN-stage, increased during secondary oocyte growth and peaked at the MAT-stage, whereas those of *cyp17a1* and *hsd3b* increased progressively from the PN- to the MAT-stage. These profiles are consistent with the dynamic changes reported in other salmonids during oogenesis [23], [31], [32]. We previously found that Fsh elevates transcripts for *star* and *hsd3b*, and to a lesser extent, *cyp11a1* and *cyp17a1* in salmon ovarian follicles [17] when plasma levels of Fsh and E2 naturally increase in this species [6], [33]. These results support the role of Fsh in ovarian steroidogenesis during early secondary oocyte growth.

During final oocyte maturation, ovarian steroidogenesis shifts from the synthesis of E2 to the maturation-inducing steroid 17α, 20β-dihydroxy-4-pregnen-3-one (17,20βP) production, and this shift is in part regulated by a surge in Lh prior to ovulation [8], [34]. At this stage, the increase of all steroidogenesis-related gene transcripts (other than *cyp19a1a*) suggests that a general enhancement of steroidogenic activity is necessary to support the rapid periovulatory increase in 17,20βP. It should be noted, however, that the marked increases in *star* and *cyp11a1* transcripts at the MAT-stage (4- and 8-fold relative to the preceding VIT-stage) and their strong and positive correlation with *lhcgr* transcripts (P<0.0001, Table 2) suggest that the delivery of cholesterol and its conversion to pregnenolone are particularly upregulated probably via Lh signaling at this stage. Similar increases in ovarian *star* and/or *cyp11a1* during maturation were found in trout [23], [31] and European sea bass [24], as well as in artificially-induced, maturing Japanese eel [35].

**Table 2:** Correlation coefficients among relative gene expression of gonadotropin receptors (*fshr* and *lhcgr*) and Fsh-regulated genes of ovarian follicles collected across stages of oogenesis in coho salmon.

	Gonadotropin receptor		Steroidogenesis					Cell survival				ECM components			
	*fshr*	*lhcgr*	*star*	*cyp11a1*	*cyp17a1*	*hsd3b*	*cyp19a1a*	*clu1*	*clu2*	*ivns1abpa*	*ddit4l*	*col1a1*	*col1a2*	*dcn*	*fn1*
***fshr***			−0.146	−0.263	0.244	0.334	0.884^c^	0.008	0.068	0.393	−0.299	0.836^c^	0.796^c^	0.792^c^	0.240
***lhcgr***	−0.149		0.989^c^	0.840^c^	0.774^b^	0.642^a^	0.036	−0.436	−0.497	−0.492	−0.682^a^	0.115	0.092	0.411	0.764^b^

	ECM remodeling		Cell proliferation				Growth factor signaling				Cell differentiation and growth	
	*ctgf*	*wapl*	*pim1*	*pcna*	*mcm4*	*tob1*	*bmp16*	*igf2*	*inha*	*smad5l*	*wt2l*	*adh8l*
***fshr***	−0.001	−0.187	−0.036	−0.164	−0.206	0.137	−0.293	0.288	0.837^c^	−0.166	0.829^c^	0.884^c^
***lhcgr***	0.668^a^	0.990^c^	−0.411	−0.418	−0.609^a^	0.070	0.982^c^	0.445	−0.040	0.762^b^	0.273	0.047

a *P*<0.01.

b *P*<0.001.

c *P*<0.0001.

Although significant in vitro effects of Fsh on *cyp19a1a* expression during early secondary oocyte growth were not previously found in salmon [17], [18], we evaluated the temporal transcript profile for this gene due its well documented increase during vitellogenic growth in other fish species [24], [36], [37]. We found that transcripts for *cyp19a1a* remained low during early secondary growth, and peaked at the VIT-stage (Fig. 2). This temporal profile showed a positive correlation with *fshr* transcripts (P<0.0001, Table 2) and correlated well with the temporal pattern of plasma E2 in coho salmon and other salmonids [8], [28], [29], supporting the idea that ovarian production of E2 is primarily stimulated by Fsh via upregulation of *cyp19a1a* mRNA and Cyp19a1a (aromatase) activity [13].

### Cell survival

**Figure 3:**
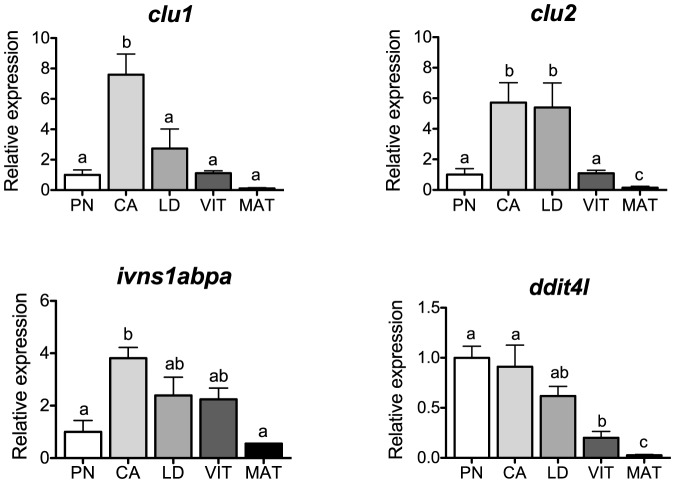
Expression profiles of genes associated with cell survival during major stages of oogenesis in coho salmon. Messenger RNA levels were analyzed by qPCR and data were normalized to the geometric mean of four reference genes (*eef1a*, *ctsd*, *ctsz* and *actb*). PN, perinucleolus stage follicles; CA, cortical alveolus stage follicles; LD, lipid droplets stage follicles; VIT, mid-vitellogenic stage follicles; MAT, postvitellogenic/preovulatory stage follicles. Bars not sharing the same letter are significantly different (P<0.05; n = 4 fish/stage for PN-, CA-, LD-, and VIT-stages, n = 3 fish for MAT-stage, mean ± SEM).

Levels of transcripts for genes associated with cell survival, *clu1*, *clu2, ivns1abpa* and *ddit4l* are shown in Fig. 3. Clusterin is a disulfide-linked heterodimeric protein with cytoprotective and anti-apoptotic properties in the ovary of chicken and rat [38], [39], although very little is known about these processes during ovarian development in fishes. Transcripts for both *clu1* and *clu2* increased in CA-stage follicles of salmon and declined toward maturation. This finding agrees with our previous study where Fsh upregulated the expression of both *clu1* and *clu2* in a concentration-dependent manner in ovarian follicles at the same stage [18], suggesting an anti-apoptotic role of this gonadotropin during early secondary oocyte growth. Interestingly, CLU was also found to mediate receptor-ligand mechanisms that lead to the uptake of vitellogenins (Vtg) into growing oocytes of chicken [38]. In fishes, Vtg enters oocytes either just after the cytoplasm fills with cortical alveoli (e.g., salmonids) or at the same time cortical alveoli appear [40] and this process is likely regulated by Fsh [12]. Although a potential role of Clu in Vtg uptake during the earlier phases of vitellogenesis in salmon is plausible, it should be specifically addressed.

A similar expression pattern was found for another gene associated with cell survival, *ivns1abpa* (Fig. 3). In mouse fibroblasts, IVNS1ABP's are suggested to protect cells from apoptosis during cell growth or changes in cell shape [41]. However, to date, there have been no functional studies for these binding proteins in the ovary of any species. We previously found that Fsh upregulated transcript levels of *ivns1abpa* in salmon ovarian follicles [18], supporting the idea that Fsh may play a pro-survival role during the onset of secondary oocyte growth.

In contrast, the cell survival-associated gene *ddit4l* showed a decreasing trend during ovarian development in salmon. In human ovarian epithelial cells, overexpression of *DDIT4* (also known as *REDD1*) promoted cell proliferation and reduced apoptosis [42], and a similar function might be possible during early oocyte growth in salmon.

### ECM components

**Figure 4:**
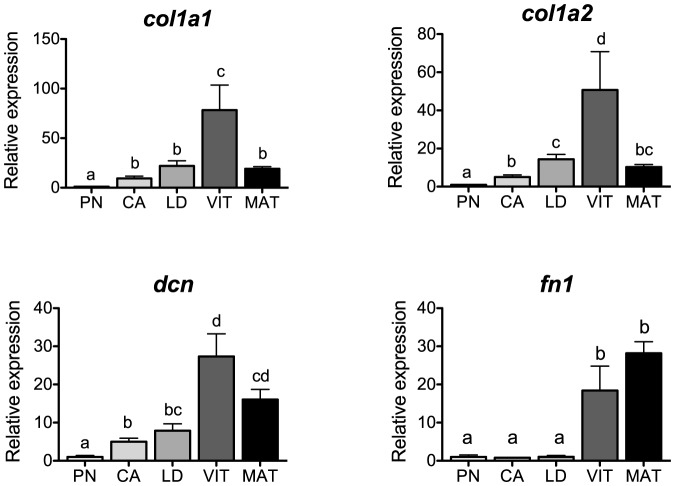
Expression profiles of extracellular matrix components during major stages of oogenesis in coho salmon. Messenger RNA levels were analyzed by qPCR and data were normalized to the geometric mean of four reference genes (*eef1a*, *ctsd*, *ctsz* and *actb*). PN, perinucleolus stage follicles; CA, cortical alveolus stage follicles; LD, lipid droplets stage follicles; VIT, mid-vitellogenic stage follicles; MAT, postvitellogenic/preovulatory stage follicles. Bars not sharing the same letter are significantly different (P<0.05; n = 4 fish/stage for PN-, CA-, LD-, and VIT-stages, n = 3 fish for MAT-stage, mean ± SEM).

Levels of transcripts for the ECM components, *col1a1* and *col1a2*, *dcn* and *fn1* are shown in Fig. 4. In mammals, the ECM provides structural support to the follicle and provides biochemical signals that promote follicle development and maturation [43]. Type-I collagen is the most ubiquitously expressed ECM molecule within the mammalian ovary [43], [44]. In salmon, levels of both *col1a1* and *col1a2* increased during secondary oocyte growth and declined at the MAT-stage, showing a strong and positive correlation with transcripts for *fshr* (P<0.0001, Table 2). As in salmon, ovarian follicular growth was also associated with an increase in levels of type-I collagen in sheep [45], whereas in medaka, ovarian *col1a1* mRNA increased as the fish matured [46]. Interestingly, type-I collagen was suggested to regulate the maintenance of E2 secretion in ovine granulosa cells [44]. In salmon, plasma levels of E2 correlate well with the stage-specific profile of *col1a1* and *col1a2* suggesting that a similarly mediated mechanism could be present.

Decorin is a small leucine-rich proteoglycan that binds type-I collagen and plays roles in a number of processes, including ECM assembly, cell cycle regulation, and inhibition of cell growth [47]. Little is known however about its specific function in ovarian biology. In the present study, *dcn* transcripts followed a similar profile as that of the collagen genes, which may indicate that these components of the ECM act together during oogenesis in salmon. Levels of *dcn* transcripts were also positively correlated with those of *fshr* (P<0.0001, Table 2), suggesting that Fsh participates in the regulation of these genes during secondary oocyte growth.

Fibronectins bind ECM components such as collagens and proteoglycans, and are often associated with cell adhesion, maintenance of cell shape, differentiation and growth [48]. Transcript levels for *fn1* remained low during previtellogenic stages, increased during vitellogenesis and peaked at the MAT-stage. This pattern was positively correlated with transcript levels of *lhcgr* (P<0.001, Table 2). In ovaries of sheep, levels of FN also increased during follicular growth [45]. In our previous study, Fsh upregulated *fn1* mRNA in the salmon ovary in vitro [18], whereas LH appeared to regulate FN in hen granulosa cells [49]. Based on the profile obtained in this study, a potential stimulatory role of both Fsh and Lh on *fn1* seems probable in the salmon ovary. It is interesting to note that treatments with FN reduced E2 secretion in ovine granulosa cells [44]. Given that *fn1* reaches maximum levels at the MAT-stage in salmon, when levels of E2 naturally decrease, a similar inhibitory mechanism on E2 production could be possible in this species.

### Tissue or ECM remodeling

**Figure 5:**
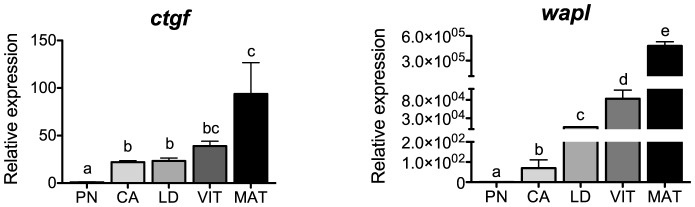
Expression profiles of genes associated with tissue or extracellular matrix remodeling during major stages of oogenesis in coho salmon. Messenger RNA levels were analyzed by qPCR and data were normalized to the geometric mean of four reference genes (*eef1a*, *ctsd*, *ctsz* and *actb*). PN, perinucleolus stage follicles; CA, cortical alveolus stage follicles; LD, lipid droplet stage follicles; VIT, mid-vitellogenic stage follicles; MAT, postvitellogenic/preovulatory stage follicles. Bars not sharing the same letter are significantly different (P<0.05; n = 4 fish/stage for PN-, CA-, LD-, and VIT-stages, n = 3 fish for MAT-stage, mean ± SEM).

Transcripts for the tissue or ECM remodeling genes, *ctgf* and *wapl*, are shown in Fig. 5. Levels of *ctgf* increased in the transition from the PN- to CA-stage, remained elevated during vitellogenesis and peaked in MAT-stage follicles, showing a positive correlation with *lhcgr* transcripts (P<0.01, Table 2). Although no definitive function for CTGF is established in vertebrate ovaries, an active role in follicle development and maturation has been suggested [50], [51]. In avian granulosa cells, expression of CTGF increased as follicular development proceeded [52], whereas in mammalian ovaries, CTGF also increased during folliculogenesis but decreased in preovulatory follicles [51], [53]. Our temporal profile in salmon, however, contrasts with a previous study where Fsh inhibited ovarian expression of *ctgf* in vitro [18]. Based on studies in hen and pig, other factor(s) such as transforming growth factor beta-1 [52] or activin and growth differentiation factor 9 [53] may upregulate the expression of *ctgf* during oogenesis in salmon.

A group of Wap domain-containing genes was characterized in brook trout ovaries and found to increase dramatically at the time of ovulation [54],[55]. In these studies, *wapl* was suggested to be involved in ovarian tissue remodeling during ovulation and cytoprotection of ovulated eggs. In agreement with these studies, transcripts for *wapl* increased during oogenesis in salmon and peaked in MAT-stage follicles (Fig. 5), showing a strong correlation with transcripts for *lhcgr* (P<0.0001, Table 2). We previously reported that Fsh also stimulated transcript levels of *wapl* during the cortical alveolus stage in vitro [18]. Although the biological implications of these findings are unknown, a potential stimulatory role of both gonadotropins on *wapl* transcription during oogenesis seems likely.

### Cell proliferation

**Figure 6:**
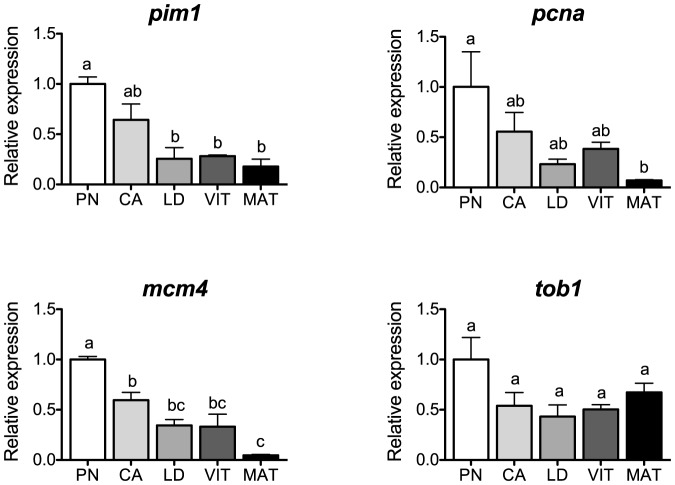
Expression profiles of genes associated with cell proliferation during major stages of oogenesis in coho salmon. Messenger RNA levels were analyzed by qPCR and data were normalized to the geometric mean of four reference genes (*eef1a*, *ctsd*, *ctsz* and *actb*). PN, perinucleolus stage follicles; CA, cortical alveolus stage follicles; LD, lipid droplet stage follicles; VIT, mid-vitellogenic stage follicles; MAT, postvitellogenic/preovulatory stage follicles. Bars not sharing the same letter are significantly different (P<0.05; n = 4 fish/stage for PN-, CA-, LD-, and VIT-stages, n = 3 fish for MAT-stage, mean ± SEM).

Transcripts for the cell proliferation genes *pim1*, *pcna*, *mcm4* and *tob1* are shown in Fig. 6. Levels of *pim1*, *pcna* and *mcm4* showed a similar stage-dependent profile characterized by high levels at the PN-stage and a steady decline toward maturation, suggesting that these transcripts are either functionally important during the PN-stage or deposited in the oocytes to play a maternal role during early embryogenesis [56]. Unfortunately, very little is known about these transcripts at the ovarian level in teleost fishes. PIM1 is a proto-oncogene involved in cell cycle progression, apoptosis and transcription activation, as well as more general signal transduction pathways [57]. In the mouse, PIM1 has been implicated in the regulation of vasculogenesis and angiogenesis of the ovary [58]. In human ovarian granulosa cells, expression of *PIM1* was inhibited by gonadotropins [59], and this finding is consistent with the downregulation of ovarian *pim1* by Fsh in vitro in salmon [18]. Results from the present study may support potential inhibition of *pim1* by both gonadotropins in the salmon ovary, although the functional relevance of this remains unknown.

PCNA is a protein intimately involved in the control of DNA synthesis and repair during cell division that regulates primordial follicle assembly in fetal and neonatal mouse ovarian follicles [60]. Somewhat in disagreement with our results, the follicle epithelium in zebrafish showed an increasing number of Pcna-positive cells during oocyte development with a maximum in vitellogenic follicles [61], although discrepancies could be due to differences in mRNA and protein synthesis. Differences in the temporal profile and localization of ovarian *PCNA* mRNA are also found in mammals [60], suggesting that regulatory mechanisms for *PCNA* may vary significantly among species.

MCM family proteins are required for initiation of DNA replication, and thus are important for cell proliferation [62]. No studies describing the potential role of MCM family proteins in the ovary of fishes or any other vertebrate species have been reported to date.

In contrast, transcript levels for the cell proliferation-related gene *tob1* did not change significantly among stages of oogenesis in salmon. The protein TOB1 has been shown to have anti-proliferative activity in a variety of cell types and also plays a role in mRNA transcription [63]. In human granulosa cells, both FSH and LH upregulated the expression of *TOB1* mRNA in vitro [59]. Fsh also enhanced the expression of *tob1* in follicle cell enriched preparations in salmon, but did not affect levels in whole ovarian follicles [18]. Considering these findings, it is possible that *tob1* is primarily regulated at the follicle cell level in the salmon ovary during oogenesis, which would explain the lack of differences in the present study where whole ovarian follicles were used.

### Growth factor signaling

**Figure 7:**
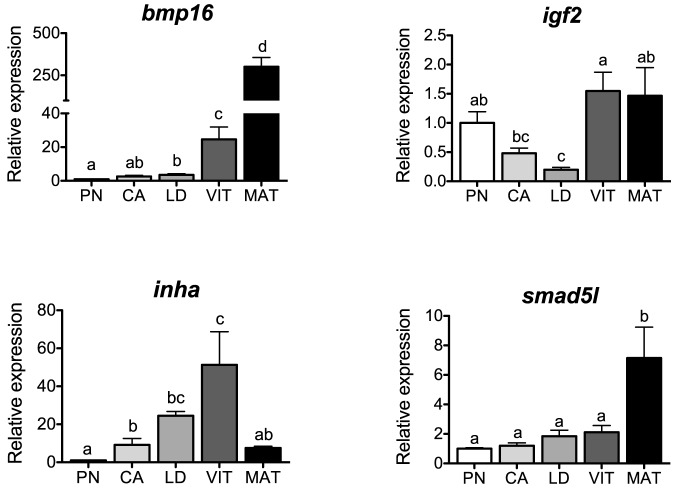
Expression profiles of genes associated with growth factor signaling during major stages of oogenesis in coho salmon. Messenger RNA levels were analyzed by qPCR and data were normalized to the geometric mean of four reference genes (*eef1a*, *ctsd*, *ctsz* and *actb*). PN, perinucleolus stage follicles; CA, cortical alveolus stage follicles; LD, lipid droplet stage follicles; VIT, mid-vitellogenic stage follicles; MAT, postvitellogenic/preovulatory stage follicles. Bars not sharing the same letter are significantly different (P<0.05; n = 4 fish/stage for PN-, CA-, LD-, and VIT-stages, n = 3 fish for MAT-stage, mean ± SEM).

Levels of transcripts for the growth factor signaling-associated genes *bmp16, igf2, inha* and *smad5l* are shown in Fig. 7. BMP's control granulosa cell proliferation and cytodifferentiation, as well as oocyte development in the mammalian ovary [64], although specific roles during fish oogenesis are unclear. In salmon, transcripts for *bmp16* increased during secondary oocyte growth and peaked at the MAT-stage (12-fold compared to the previous VIT-stage), showing a strong and positive correlation with *lhcgr* transcripts (P<0.0001, Table 2). Recently, we found that Fsh suppressed transcripts for *bmp16* during the cortical alveolus stage in vitro [17]. Results from the present study indicate that Bmp16 plays a primary role during final oocyte maturation in salmon. In a previous study, expression of two Bmp receptors (*bmpr2a* and *bmpr2b*) increased during folliculogenesis in zebrafish and peaked in full-grown oocytes, suggesting a role for Bmp's in the control of final maturation and ovulation in this species [65]. However, some dissimilarities are observed with other members of the Bmp family in fishes. For example, expression of *bmp4* and *bmp7* were the highest during the pre-vitellogenic stages in rainbow trout and decreased with advancement of follicle stage [66]. A similar tendency was observed for *bmp4* and *bmp15* in European sea bass [67]. In contrast, *bmp15* levels did not change during follicular growth in zebrafish [68]. These discrepancies suggest different roles of Bmp family members during oogenesis in fishes.

IGF2 promotes cell growth and proliferation in a variety of cell types, including mammalian granulosa cells [69]. In the present study, transcripts for *igf2* declined during the transition to secondary oocyte growth and increased at the VIT- and MAT-stages. In agreement with this profile, levels of *igf2* also increased during oocyte maturation in rainbow trout [70] and treatments with Igf2 induced meiotic resumption and enhanced oocyte maturational competence in a number of fish species [71], [72]. In comparison, very little is known about the role of Igf2 during vitellogenesis in fishes. In coho salmon, Fsh had a slight positive effect on *igf2* transcripts in CA-stage follicles [17], and treatments with Igf2 increased DNA synthesis in vitellogenic follicles of goldfish [73]. Interestingly, IGF2 increased E2 production and *CYP19A1* mRNA levels in bovine granulosa cells [74]. Considering that levels of plasma E2 and ovarian *cyp19a1a* mRNA are the highest during vitellogenesis in salmon, a similar regulatory action of Igf2 might be present in this species.

Inhibin is an important inhibitor of pituitary FSH production through negative feedback from the gonad [75], [76]. In the present study, transcript levels for *inha* were lowest at the PN-stage, steadily increased during secondary oocyte growth, peaked during vitellogenesis and decreased in MAT-stage follicles, showing a strong and positive correlation with transcripts for *fshr* (P<0.0001, Table 2). Likewise, transcripts for *inha* increased during follicle development and dropped sharply in maturing follicles of zebrafish [76]. In this zebrafish study, Fsh but not Lh stimulated ovarian *inha* mRNA, as also reported in mammals [77]. Our results support the idea that in fishes, as in mammals, pituitary Fsh and ovarian inhibin may form a feedback loop.

SMAD's are intracellular proteins that transmit signals for TGF ligands and modulate the biological effect of BMP's. The expression of *SMAD* genes in the ovary is well documented in humans [78]. In zebrafish, transcripts for *smad2*, *3*, *4* and *7* were detected in the ovary [79], [80], and recombinant Bmp2b and Bmp4 activated Smad1/5/8 in cultured follicle cells [65]. In SMAD5 knockout mice, primordial germ cell development is impaired [78]. In the present study, transcripts for *smad5l* were low during primary and secondary oocyte growth and peaked at the MAT-stage, showing a positive correlation with transcripts for *lhcgr* (P<0.001, Table 2). These findings suggest a potential role of *smad5l* during final oocyte maturation in salmon.

### Cell differentiation and growth

**Figure 8:**
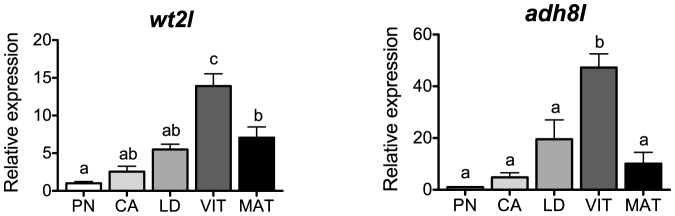
Expression profiles of genes associated with cell differentiation and growth during major stages of oogenesis in coho salmon. Messenger RNA levels were analyzed by qPCR and data were normalized to the geometric mean of four reference genes (*eef1a*, *ctsd*, *ctsz* and *actb*). PN, perinucleolus stage follicles; CA, cortical alveolus stage follicles; LD, lipid droplet stage follicles; VIT, mid-vitellogenic stage follicles; MAT, postvitellogenic/preovulatory stage follicles. Bars not sharing the same letter are significantly different (P<0.05; n = 4 fish/stage for PN-, CA-, LD-, and VIT-stages, n = 3 fish for MAT-stage, mean ± SEM).

Gene expression profiles for cell differentiation and growth related genes, *wt2l* and *adh8l*, are shown in Fig. 8. Mammalian WT's are zinc-finger transcription factors, encoded by the tumor suppressor gene *WT1*, with a potential role in the suppression of the development of immature follicles [81]. Transcripts for *WT1* have repeatedly been reported to be highest in immature follicles and decline as oogenesis proceeds in avian and mammalian species [82], [83]. In contrast, transcripts for *wt2l* in salmon were lowest at the PN-stage and steadily increased leading up to vitellogenesis, showing a positive correlation with levels of *fshr* (P<0.0001, Table 2). In agreement with this profile, we previously found that Fsh stimulates transcript levels of *wt2l* in vitro [18], suggesting that expression of ovarian *wt2l* is under stimulatory control of Fsh in salmon. Given that levels of *wt2l* decrease in MAT-stage follicles, it will be interesting to investigate whether Wt2l plays any role in suppressing final maturation of the oocyte in this species.

ADH enzymes function in the metabolism of retinoic acid, which is crucial for proper embryonic development in vertebrates. Several publications have reported ovarian expression of genes involved in the retinoid pathway in fishes [84], [85] although information specifically on Adh8 is almost nonexistent. In medaka, *adh8* was detected in several tissues but not in the ovary [86]. In the present study, transcripts for *adh8l* were lowest at the PN-stage, steadily increased leading up to vitellogenesis, and decreased at the MAT-stage. This pattern was also positively correlated with the profile of *fshr* transcripts (P<0.0001, Table 2). The marked increase of *adh8l* mRNA in the salmon ovary during vitellogenic growth may be explained by the fact that retinoids are accumulated in the egg yolk during vitellogenesis in oviparous vertebrates [85], a process mainly regulated by Fsh [12]. In contrast, we previously reported that Fsh inhibited the expression *adh8l* in vitro at the cortical alveolus stage in salmon [18], suggesting that factors other than Fsh might be involved in the regulation of ovarian *adh8l* expression during secondary oocyte growth in salmon.

## Conclusions

In the present study, expression profiles of a suite of genes regulated in vitro by Fsh were characterized during oogenesis in coho salmon. We found a group of genes that increased during secondary oocyte growth and declined during maturation, showing a strong and positive correlation with ovarian *fshr* transcripts and providing further support for involvement of Fsh in the regulation of specific cellular processes. This group included genes associated with steroidogenesis (*cyp19a1a*), growth factor signaling (*inha*), cell differentiation and growth (*wt2l* and *adh8l*), and ECM components (*col1a1*, *col1a2*, and *dcn*). Other genes involved in cell survival (*clu1*, *clu2* and *ivns1abpa*), and ECM function (*fn1*) and growth factor signaling (*igf2*) seemed to play a role during early and late secondary oocyte growth, respectively. These data on gene expression profiles of Fsh-regulated genes during oogenesis in salmon provide a basis for more detailed studies of the role of Fsh in specific processes during ovarian follicle development in fishes.

## Supporting Information

S1 Figure **Relative expression of reference genes and their geometric mean during major stages of oogenesis in coho salmon.** Messenger RNA levels were analyzed by qPCR. PN, perinucleolus stage follicles; CA, cortical alveolus stage follicles; LD, lipid droplet stage follicles; VIT, mid-vitellogenic stage follicles; MAT, postvitellogenic/preovulatory stage follicles. Bars not sharing the same letter are significantly different (P<0.05; n = 4 fish/stage for PN-, CA-, LD-, and VIT-stages, n = 3 fish for MAT-stage, mean ± SEM).
(EPS)
